# Construction of miRNA-mRNA network for the identification of key biological markers and their associated pathways in IgA nephropathy by employing the integrated bioinformatics analysis

**DOI:** 10.1016/j.sjbs.2021.06.079

**Published:** 2021-07-01

**Authors:** Fatima Noor, Muhammad Hamzah Saleem, Muhammad Farhan Aslam, Ajaz Ahmad, Sidra Aslam

**Affiliations:** a Department of Bioinformatics and Biotechnology, Government College University, Allama Iqbal Road, 38000 Faisalabad, Pakistan; b MOA Key Laboratory of Crop Ecophysiology and Farming System in the Middle Reaches of the Yangtze River, College of Plant Science and Technology, Huazhong Agricultural University, Wuhan 430070, China; c School of Biological Sciences, University of Edinburgh, United Kingdom; d Department of Clinical Pharmacy, College of Pharmacy, King Saud University, Riyadh 11451, Saudi Arabia

**Keywords:** Bioinformatics analysis, Immunoglobulin A nephropathy, Gene expression profiling, Protein-protein interaction, Hub genes, Hub genes-miRNA network, IgAN, Differential Expressed Genes, PPI, Immunoglobulin A nephropathy, miRNA, Protein-Protein Interaction, GEO, MicroRNA, DAVID, Gene Expression Omnibus, GO, Database for annotation visualizationand integrated discovery, KEGG, Gene Ontology, MF, Kyoto Encyclopedia of Genes and Genomes, CC, Molecular function, BP, Cellular components, STRING, Biological process, MCODE, Search Tool for the Retrieval of Interacting Genes/Proteins, ENCORI, Molecular Complex Detection

## Abstract

**Background:**

About half-century ago, Immunoglobulin A nephropathy (IgAN) was discovered as a complicated disease with frequent clinical symptoms. Until now, exact mechanism underlying the pathogenesis of IgAN is poorly known. Therefore, current study was aimed to understand the molecular mechanism of IgAN by identifying the key miRNAs and their targeted hub genes. The key miRNAs might contribute to the diagnosis and therapy of IgAN, and could turn out to be a new star in the field of IgAN.

**Methods:**

The microarray datasets were downloaded from Gene Expresssion Omnibus (GEO) database and analyzed using R package (LIMMA) in order to obtain differential expressed genes (DEGs). Then, the hub genes were identified using cytoHubba plugin of cytoscpae tool and other bioinformatics approaches including protein-protein interaction (PPI) network analysis, module analysis, and miRNA-hub gene network construction was also performed.

**Results:**

A total of 348 DEGs were identified, of which 107 were upregulated genes and 241 were downregulated genes. Subsequently, the 12 overlapped genes were predicted from cytoHubba, and considered as hub genes. Moreover, a network among miRNA-hub genes was created to explore the correlation between the hub genes and their targeted miRNAs. Network construction ultimately lead to the identification of nine gene named FN1, EGR1, FOS, JUN, SERPINE1, MMP2, ATF3, MYC, and IL1B and one novel key miRNA namely, has-miR-144-3p as biomarker for diagnosis and therapy of IgAN.

**Conclusion:**

This study updates the information and yield a new perspective in context of understanding the pathogenesis and development of IgAN. In future, key miRNAs might be capable of improving the personalized detection and therapies for IgAN. *In vivo* and *in vitro* investigation of miRNAs and pathway interaction is essential to delineate the specific roles of the novel miRNAs, which may help to further reveal the mechanisms underlying IgAN.

## 1 Introduction

The most common glomerular disease, Immunoglobulin A nephropathy (IgAN) was first uncovered by Jacques Berger about half a century ago (Berger, 1968, Hu et al., 2020). IgAN is arrived as an important issue for health care (Schena, 1990). IgAN is manifested by the deposition of Immunoglobulin A in glomerulus. However, the exact pathogenesis is little known (McGrogan et al., 2011). It has a great diversity of clinical symptoms that vary widely in terms of disease status and prognosis. Apoptosis (Liang et al., 2017, Leung et al., 2015), cell proliferation (Zhang et al., 2017, Rops et al., 2018), sustained inflammation (Rauen and Floege, 2017) and fibrosis (Hennino et al., 2016, Tanaka et al., 2018) are responsible for the pathogenesis of IgAN. Different signaling pathways and genes e.g, those encoding the Tank binding kinase 1(TBK1) (Qian et al., 2019), transforming growth factor-(TGF-β) (Lim et al., 2005), Megsin (Yating et al., 2017), etc. are involved in the development of IgAN. However, owing to insufficient diagnostic methods, IgAN patients are diagnosed, on average, at middle or late disease stage (Qian et al., 2019), which consequently, leads to the poor prognosis. Hence, the understanding of molecular mechanisms contributes to the pathogenesis and prognosis of IgAN has become increasingly important for the development of multiple therapeutics and diagnostics approaches.

The discovery of potential biomarkers that can halt the pathophysiology of the disease and can act as a virtual shortcut, will considered as the miracle of the current era. Mind boggling potential benefits of molecular biomarkers offers multiple innovative perspective to improve diagnostic as well as treatment option. Micro-RNAs (miRNAs) are small non-coding RNA molecules, involved in the post-transcriptional gene expression of countless metabolic pathways (Zhang et al., 2006). Multiple studies have shown that miRNAs may have a critical role to play in pathogenesis of human diseases including IgAN (Bartels and Tsongalis, 2009, Xiao and Rajewsky, 2009). In recent decades, bioinformatics analysis and microarray technology enable researchers to identify the miRNA involved in the pathogenesis of IgAN.

In spite of the numerous studies on autoimmune diseases, no sufficient evidence is present yet to prove the existence of miRNAs, and their involvement in the pathogenesis and development of IgAN. To tackle this issue, we used integrated bioinformatics approaches to figure out the disease-related gene and their targeted novel miRNAs as problem-solving negotiators to switch off the progression of IgAN. Moreover, identification of hub genes and their associated miRNAs might consider as novel diagnostic and therapeutic biomarkers for IgAN. Moreover, investigation of mRNA-miRNA interactions in the present work can contribute to the discovery of therapeutic candidates. Lastly, we compare our findings to previous research in order to better understand the molecular mechanism of IgAN.

## 2 Materials and methods

### 2.1 Retrieval of data

Gene Expression Omnibus (GEO) database in National Center for Biotechnology Information (NCBI) is a freely available public database, enclosing the gene profiles. By using the Human IgAN as a search term, microarray datasets (GSE93798) were retrieved from NCBI-GEO database (Barrett et al., 2012). The microarray profile dataset GSE93798 was obtained from Affymetrix’s HGU133 Plus 2 chip comprised of 22 healthy controls and 20 IgAN patients.

### 2.2 Identification of DEGs

Differentially Expressed Genes (DEGs) between normal subjects and IgAN patients were identified using LIMMA package (Ritchie et al., 2015). Limma is an R/Bioconductor software package that provides an integrated solution for analysing data from gene expression experiments.   Later, genes that satisfy the criteria of |log fold change (FC)| > 1.0 and adjusted P-value < 0.01 were distinguished as DEGs. Volcano plot were constructed using ggplot2 package available in R, to visualize the significant and non-significant DEGs.

### 2.3 Analysis of DEGs at functional level

At functional level, database for annotation, visualization, and integrated discovery (DAVID) was used to perform GO enrichment analysis and KEGG pathway analysis. The DEGs were subjected to DAVID for the prediction of the function of DEGs at three level: Molecular function (MF), Biological process (BP), and Cellular component (CC). The top 10 significant items of GO and KEGG pathways were demonstrated in form of bubble maps. Using ggplot2 package available in R, a bubble map was constructed on the basis of P value. In this regard, P < 0.05 was believed to be statistically-significant.

### 2.4 Construction of protein-protein interaction network

Protein-protein interaction (PPI) network were constructed to determine the functional interactions between the resulted DEGs. Search Tool for the Retrieval of Interacting Genes/Proteins (STRING) was used for the functional interactions among DEGs at the combined score of >0.7 (Mering et al., 2003). The resulted genes were subjected to the Cytoscape_v3.8.2 (Shannon et al., 2003). Molecular Complex Detection (MCODE) plugin from cytoscape was utilized for distinguishing the module that best represent the clusters of DEGs (Cline et al., 2007). In MCODE, the modules were considered significant having number of nodes >5 and the score ≥5. In the current work, the 3 topmost modules were considered significant as the node number >5 and score ≥5. Further, the resulted three modules were subjected to DAVID for the KEGG pathway analysis.

### 2.5 Selection of hub genes

CytoHubba plugin was used for distinguishing hub genes among DEGs. A total of twelve topological analysis methods are available in the cytoHubba. Among the 12 methods, MNC, degree, betweenness, and closeness methods were choosed for the identification of hub genes. Later, the topmost twenty-five genes ranked by MNC, degree, betweenness, and closeness were selected. Finally, the Venn diagrams tools was used for the identification of overlapped genes, considered as hub genes.

### 2.6 Construction of miRNA-hub genes network

**Fig. 1:**
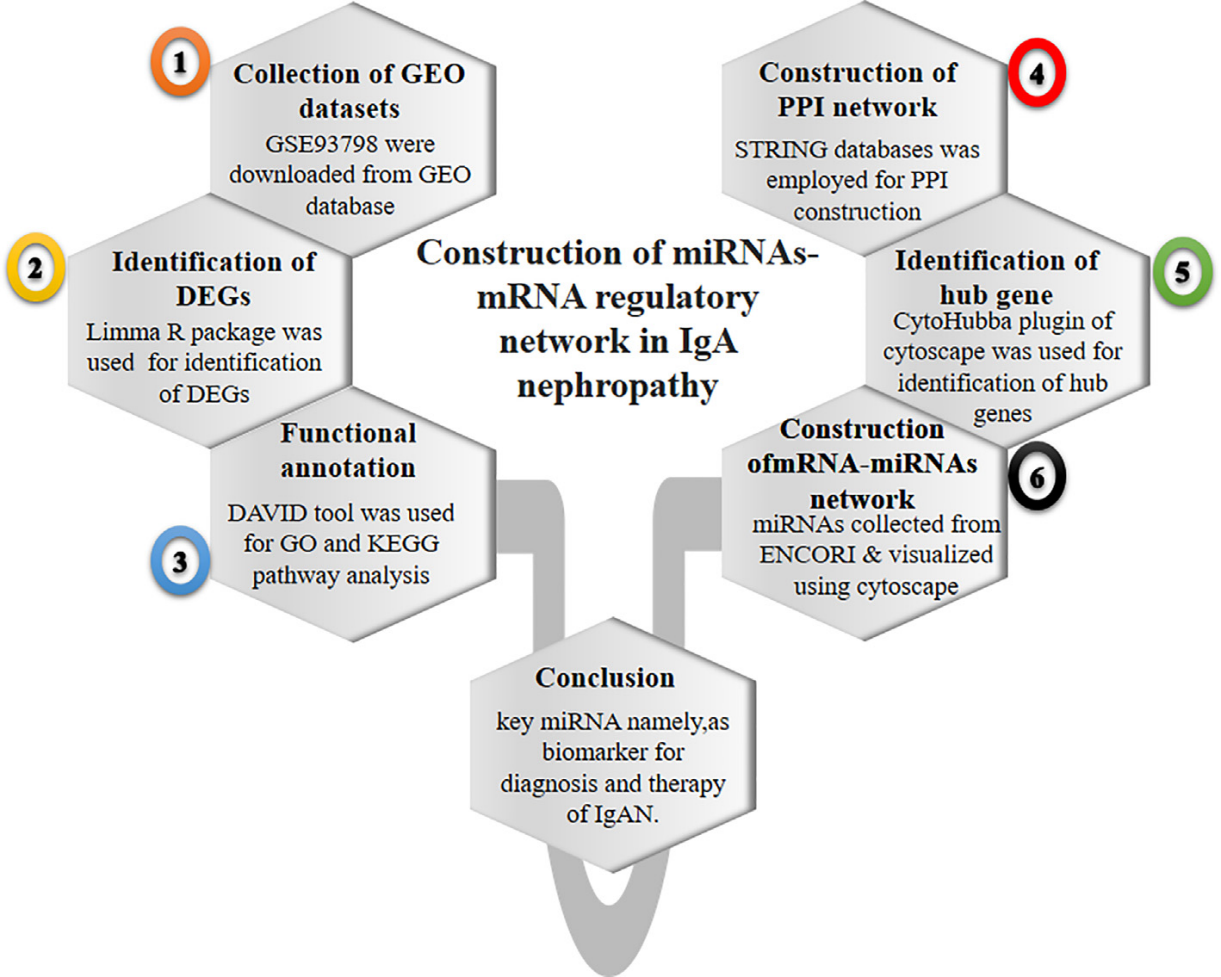
Graphical synopsis of representing the overall strategy used in the prediction key miRNAs as potential biomarker.

For miRNA-hub genes interaction, Encyclopedia of RNA interactomes (ENCORI) was used, which integrates 8 different databases for the prediction of miRNA- targets. In present work, four databases namely Tragetscan, PITA, PicTar, and miRanda were used for the prediction of miRNA- targets. The miRNA was considered as targeted miRNA of that particular hub genes, if the resulted miRNA were present in at least 2 databases (Tragetscan, PITA, PicTar, and miRanda). Furthermore, cytoscape software was used to construct a visualization interaction network among targeted miRNA and hub genes. Moreover, the overall methodology that are used in the cureent analysis is outlined in Fig. 1.

## 3 Results

### 3.1 Identification of DEGs

**Fig. 2:**
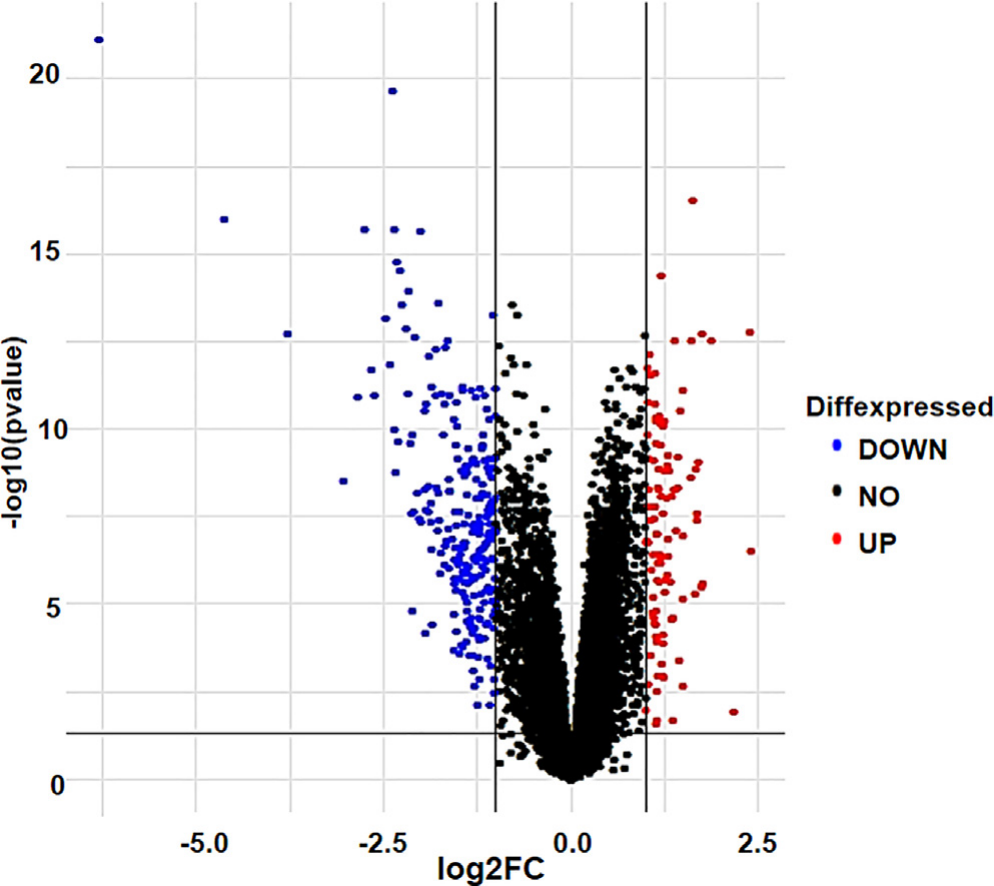
Representation of differential expressed genes in form of Volcano plot with red dots showing upregulated genes while blue dots showing downregulated genes. Black dots represent non-significant genes.

In the present study, microarray dataset GSE93798 was obtained from Affymetrix’s HGU133 Plus 2 chip. GSE93798 consists of 42 samples (22 healthy controls and 20 IgAN patients). Total of 348 significant DEGs (Supplementary file 1) were obtained from datasets including 107 upregulated and 241 downregulated genes (Table 1). Results of the expression level analysis are presented in a volcano plot (Fig. 2).

**Table 1:** A total 348 DEGs were identified of which 107 upregulated and 241 downregulated genes.

Differential Expressed Genes (DEGs)	Name of the genes
*Upregulated genes*	
(107)	PCDH18, LYL1, COL1A2, SOX17, C8orf4, FPR3, GATA3, EMP3, GUCY1A3, FXYD5, NETO2, CD44, PTGS1, SYNPO2, SRPX2, FN1, SYT11, COL5A2, CYSLTR1, SNAI2, MECOM, TGFBI, MARCKS, MMP2, GPR65, HLX, CRTAM, SGK223, CDO1, ECM1, C3AR1, RTN1, RRM2, TOP2A, TWIST1, APOC1, CD14, NRARP, MN1, CSF1R, FNDC1, LINC01279, COL3A1, LPAR6, CLEC5A, AEBP1, SOX18, IL33, CCL8, COL1A1, UCP2, CRIP1, COL15A1, TMEM200A, PLA2G4A, OLFML3, CTHRC1, SFRP2, KIAA0922, HCLS1, PLEK, LUM, HHEX, NDC80, TYROBP, IL10RA, MOXD1, IFI30, GJA4, CYBB, UCHL1, C1QA, PLA2G7, VSIG4, HTR2B, TAC1, MPEG1, C15orf48, MS4A7, COL6A3, POSTN, CD36, C1QB, THBS2, CX3CR1, OMD, APLNR, LYZ, IL1B, SUCNR1, COL21A1, UBD, LOC101927451, IGSF6, CHODL, CXCL11, IGHM, IDO1, COLEC12, HBB, CCL4, CXCL10, ADH1B, HLA-DQA1, IGKC, JCHAIN, SELE
*Downregulated genes*	
(241)	FOSB, DUSP1, FOS, ZFP36, EGR1, RNF186, CEBPD, JUN, CSRNP1, ERRFI1, CYP27B1, PPP1R10, DEPDC7, KLF4, APOLD1, ATF3, SLC19A2, PER1, TIPARP, JUNB, NR4A2, RASD1, KLF6, GDF15, MAOA, PDE4B, BTG2, GSTA3, BHLHE40, NR4A1, KLF9, PHLDA1, BRE-AS1, KCNK5, CD69, RASL11B, SPRY2, PCK1, NFIL3, RDH10, PDK4, CTH, ATOH8, NR1H4, CRYM, LINC00473, ESM1, PLD6, NFKBIZ, IP6K3, GSTA1, CPNE4, RUNDC3B, SOWAHC, AK4, ALDH6A1, ZNF331, SLC23A1, C11orf54, DDC, ARG2, LRP2, CMBL, ANKRD33B, GADD45B, VNN1, ID4, WDR72, NR4A3, HPD, MGST1, FOSL2, UGT2A3, TSC22D3, CEBPB, LOC727944, CXCL2, SHMT1, ACMSD, TNFAIP3, SLC7A9, LRRC19, ALDH8A1, DNAJB1, FABP1, SULT1C2, ASS1, ANK2, BHMT2, GIPC2, SH3GL2, NOX4, ESRRG, NAPSA, SLC2A2, KHK, BAG3, ZNF189, PBLD, MT1F, MAFF, DUSP6, ALDOB, SLC17A3, ALB, GPAT3, PHACTR3, SLC17A1, HGD, TMEM252, SDC1, SLC27A2, AFM, DIO1, DPYS, APOM, MT1X, PPP1R15A, SLCO4C1, DDIT4, MRO, CYP8B1, RBP5, TMEM27, AZGP1, A1CF, SLC22A11, SLC13A3, FBP1, IER2, SLC4A4, CYR61, PPARGC1A, SLC16A9, G6PC, ARRDC2, DMGDH, ANKS1B, SLC3A1, LOC284454, CLRN3, GATM, DUSP2, BBOX1, XPNPEP2, ARC, FOXQ1, MIOX, ARG1, GADD45G, PIPOX, SLC5A12, CYP3A7, DPEP1, HRG, IMPA2, PLG, SLC22A6, ITPRIP, ENPP6, SDPR, GLYAT, SOCS3, GBA3, SMIM24, SLC13A1, C10orf10, MGAM, ACE2, PMAIP1, AGXT2, SLC47A1, AGMAT, SLC22A8, LOC100506498, TINAG, CDKN1A, SLC47A2, SMIM3, FAM150B, MYC, FREM2, BNC1, FAM151A, GLYATL1, MRLN, PAH, PSAT1, RIDA, DAO, FMO1, CUBN, KCNJ15, AGT, BHMT, NAT8, METTL7B, AKR1C1, HSPA1B, MYRIP, HBEGF, CXCR2, RGS1, EGR3, MT1M, SOX9, SERPINA1, HAO2, APOH, SERPINE1, C2CD4A, CLDN2, SLC25A18, AREG, MT1G, AOC1, FGB, TOX3, CTXN3, SLC23A3, CALB1, CRISPLD2, HSPA6, RNF212B, ERAP2, SLC10A2, CYP4F2, TMED6, S100A12, FOSL1, LOC100505985, TMEM213, ERP27, FCGR3B, S100A8, S100A2, RBP4, APOD, AKR1B10, IGFBP1, CLDN8

### 3.2 Functional enrichment analysis of DEGs

**Fig. 3:**
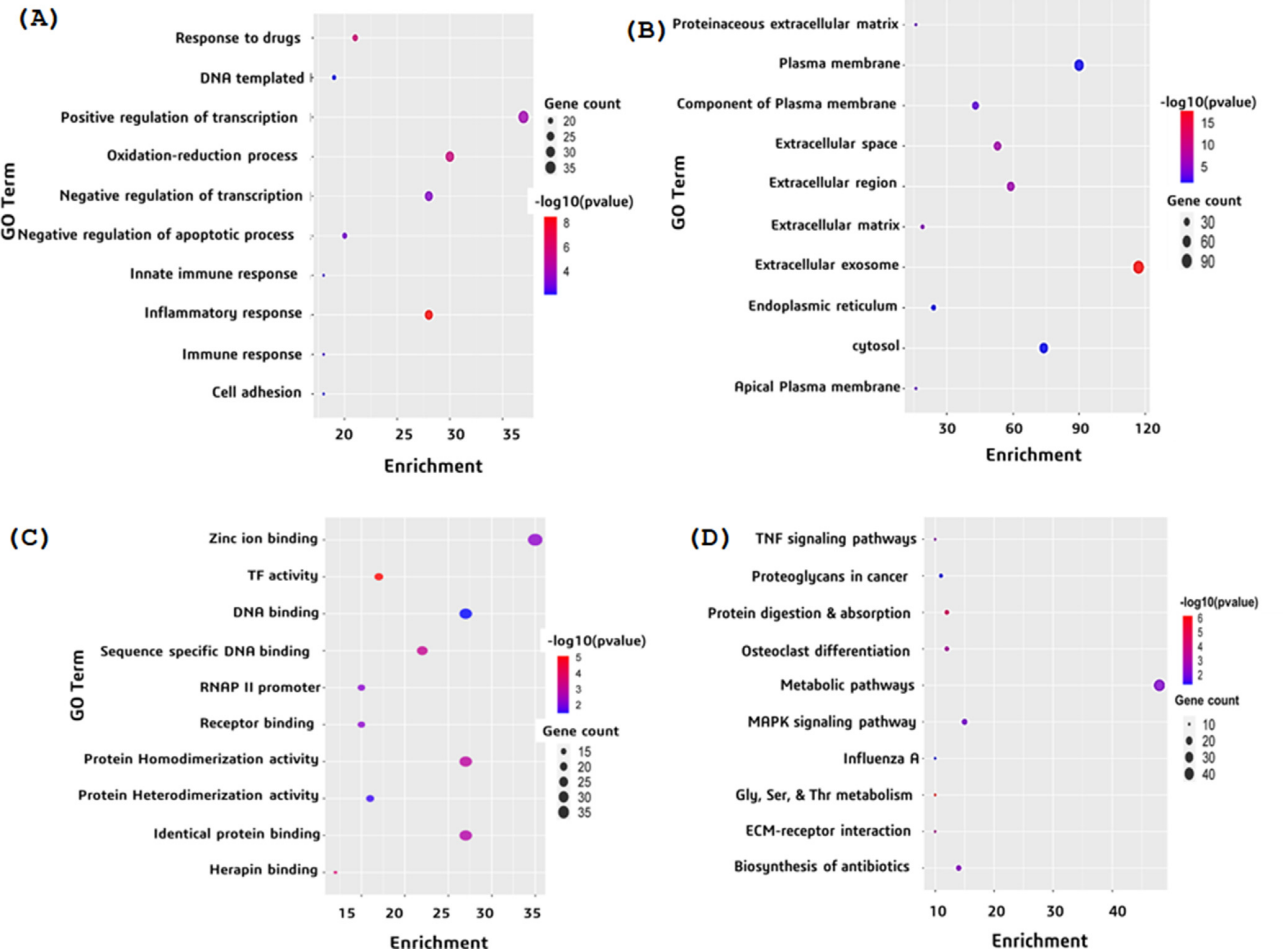
Representation of GO enrichment and KEGG pathway analysis. (a) Gene ontology in terms of Biological processes (b) Gene ontology in terms of Cellular Components (c) Gene ontology in terms of Molecular function (d) KEGG pathway analysis.

GO enrichment and KEGG pathways analysis of DEGs were performed to analyze the gene function in terms of biological processes, cellular components, and molecular function as well as their associated pathways. GO enrichment analysis of top 10 significantly enriched terms showed that in BP category, the genes involved are concerned with response to drug, positive regulation of transcription, DNA-templated, positive regulation of transcription from RNA polymerase II promoter, and negative regulation of transcription from RNA polymerase II promoter (Fig. 3(a)). In terms of CC, the genes were enriched in proteinaceous extracellular matrix, plasma membrane, integral component of plasma membrane, extracellular space, and extracellular region (Fig. 3(b)). For MF, category the genes were mainly concentrated in the zinc ion binding, transcriptional activator activity, RNA polymerase II core promoter proximal region sequence-specific binding, transcription factor activity, and sequence-specific DNA binding and RNA polymerase II core promoter proximal region sequence-specific DNA binding (Fig. 3(c)). KEGG enrichment pathway analysis revealed that genes were significantly enriched in the TNF signaling pathway, metabolic pathways, osteoclast differentiation, protein digestion and absorption, and proteoglycans in cancer (Fig. 3(d)).

### 3.3 Construction of PPI network and the analysis of DEGs

**Fig. 4:**
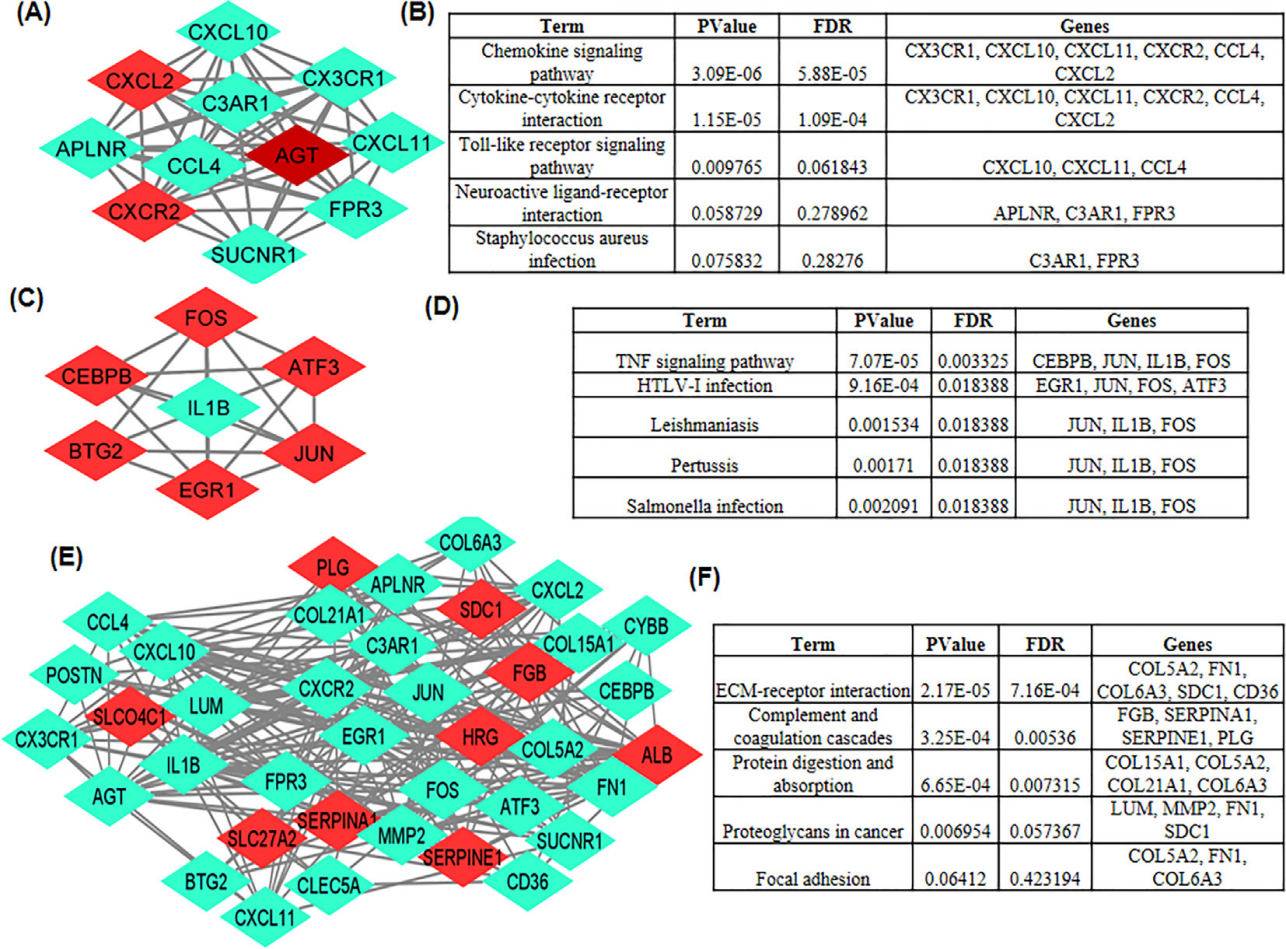
3 modules were selected having cutoff criteria node >5 and the score is ≥5. (a) First module constructed from MCDOE comprised of 11 genes. (b) Pathways associated with first module (c) Second module constructed from MCDOE comprised of 7 genes. (d) Pathways associated with the second modules. (e) Third module constructed from MCDOE comprised of 20 genes. (f) Pathways associated with the third modules.

PPI network of DEGs obtained from STRING were subjected to the MCODE plugin of cytoscape which provided significant 11 modules. From these modules, the top three functional clusters of modules were selected based on the cutoff criteria of node >5 and the score is ≥5 (Table 2). KEGG pathway analysis of the selected modules revealed that the genes belong to these clusters are enriched in cytokine-cytokine receptor interaction, toll-like receptor signaling pathway, TNF signaling pathway, chemokine signaling pathway, and complement and coagulation cascades (Fig. 4).

**Table 2:** Top 3 modules were selected having cutoff criteria node >5 and the score is ≥5.

Clusters	Score	Nodes	Edges	Nodes IDs
1	11.000	11	55	CXCL10, SUCNR1, C3AR1, CX3CR1, APLNR, AGT, CCL4, FPR3, CXCR2, CXCL2, CXCL11
2	6.000	7	18	JUN, EGR1, ATF3, IL1B, FOS, CEBPB, BTG2
3	5.474	20	52	COL6A3, HRG, SDC1, CD36, COL21A1, SERPINE1, COL15A1, SLCO4C1, ALB, SERPINA1, PLG, SLC27A2, FGB, CYBB, LUM, MMP2, POSTN, FN1, CLEC5A, COL5A2

### 3.4 Selection of hub genes

**Fig. 5:**
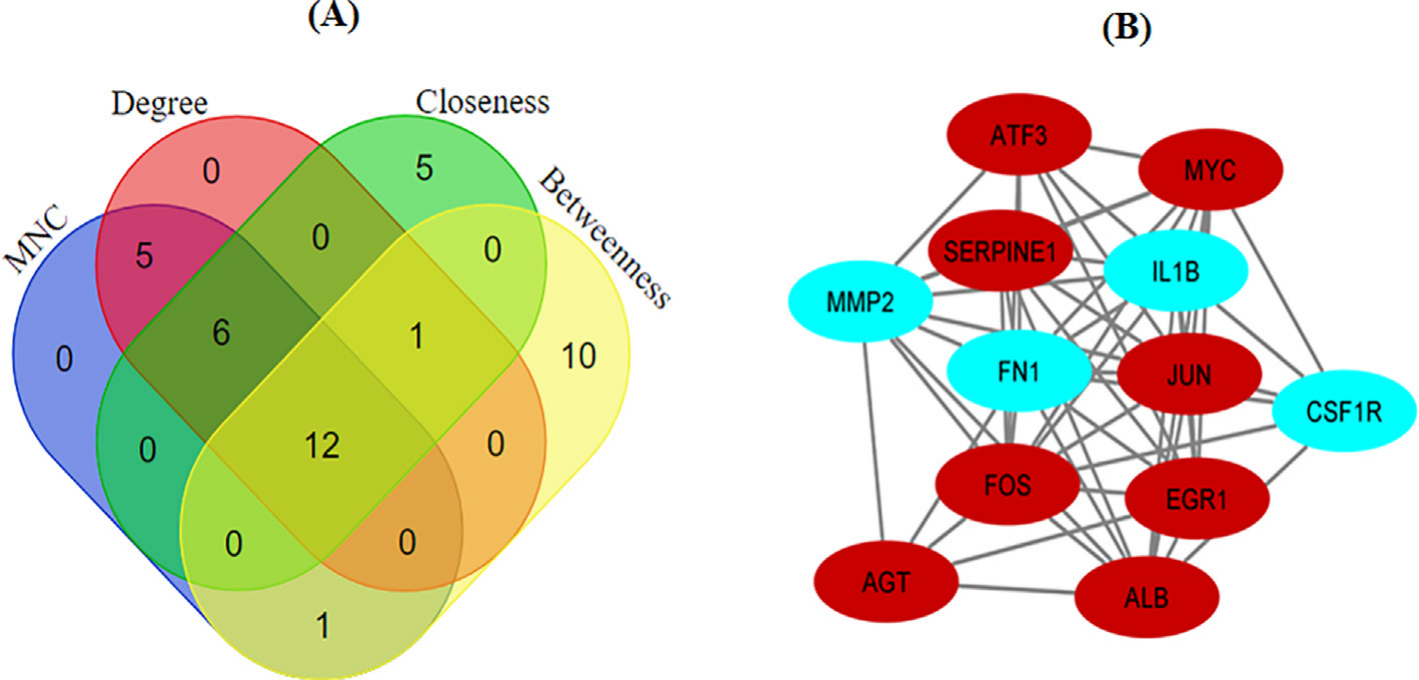
(a) Common differential expressed genes identified between 4 methods cytohubba (b) Construction of PPI network of 12 hub genes.

Using three methods available in the cytoHubba, the topmost twenty-five genes were selected and ranked by MNC, degree, betweenness, and closeness methods. The resulted genes were subjected to Venn diagrams for the identification of overlapped genes (Fig. 5). A total of 12 overlapped genes were identified, those considered as the hub genes.

### 3.5 miRNA-mRNA network

**Fig. 6:**
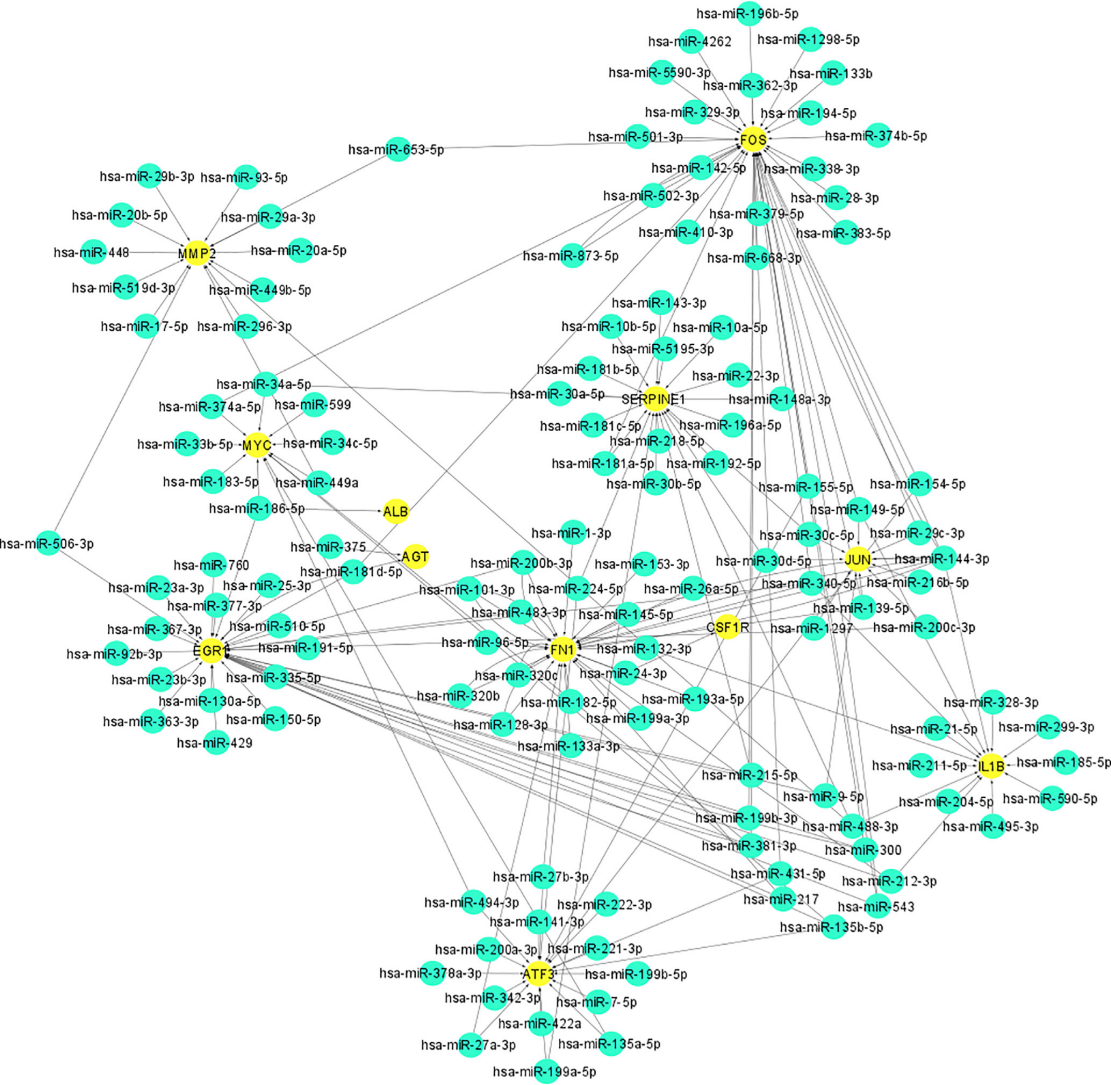
Construction of network among miRNA-hub genes from cytoscape. Yellow circles in network represent the hub genes while the blue circle in network represent miRNA followed by arrows which shows the interaction among miRNA and hub genes.

Four different databases (Tragetscan, PITA, PicTar, and miRanda) from ENCORI were used for the identification of miRNAs targeted by the hub genes. In this regard, miRNA-target network was constructed in order to visualize the interaction between miRNA-hub genes by using Cytoscape software (Fig. 6). The miRNA was considered as targeted miRNA of that particular hub genes, if the resulted miRNA were present in at least 2 databases (Tragetscan, PITA, PicTar, and miRanda) (Supplementary file 2). Solid black lines represent the interaction between hub genes with their corresponding targeted miRNA. Using degree method in cytoHubba plugin, the topmost 10 molecules were selected. From cytoHubba, it has been estimated that FN1 (degree score = 56), EGR1 (degree score = 54), FOS (degree score = 49), JUN, (degree score = 36), SERPINE1 (degree score = 35), and MMP2 (degree score = 26) are 6 interactional hub genes that have been intended to target more miRNAs, followed by the ATF3 (degree score = 19), MYC (degree = 16), and IL1B (degree score = 14). Moreover hsa-miR-144-3p (degree score = 8) was the top miRNA that considered to be targeted most hub genes.

## 4 Discussion

IgAN is considered as primary renal disease worldwide and elucidates 20% to 47% of primary glomerular disease (Wyatt and Julian, 2013, Rodrigues et al., 2017). It has essentially been described by hypertension, proteinuria, hematuria, and renal dysfunction (Yu et al., 2020). IgAN is most common in Pacific and East Asian than Africans (Rodrigues et al., 2017) and considered as major cause of last-stage kidney disease. Until now, IgAN dramatically increases the morbidity as well as the mortality rate in developed countries (Jarrick et al., 2017). As the information pileup, the same scenario is predicted in the developing countries. Bioinformatics analysis offers various high throughput approaches to deal with the IgAN patient. Clinically, the more frequent indication of IgAN is hematuria (Coppo and Robert, 2020). There has been substantial heterogeneity regarding various hematuria cases that leads the earlier detection, a thought-provoking question (Barratt and Feehally, 2006, D'Amico, 1988). The current work was planned to identify the disease related functional genes along with their key miRNAs involved in the progression of IgAN, this whole research revolves around the analysis of gene ontology, gene enrichment pathways, PPI, hub genes, and mRNA-miRNAs interaction. In the current work, 12 genes were found to be altered in IgAN patient. Later, the interaction network among miRNA-hub genes revealed 10 significant nodes including FN1, EGR1, FOS, JUN, SERPINE1, MMP2, ATF3, MYC, IL1B, and has-miR-144-3p. Consequently, the present study revealed a key miRNA named has-miR-144-3p that is targeted by more than half of the hub genes. Hence, it represents that this particular miRNA has a crucial role in the pathogenesis of IgAN.

Through KEGG pathway analysis performed in DAVID, the DEGs were found to be significantly enriched in the TNF signaling pathway, metabolic pathways, osteoclast differentiation, protein digestion and absorption, proteoglycans in cancer metabolic pathways, MAPK signaling pathway, influenza A, glycine, serine and threonine metabolism, ECM-receptor interaction, and biosynthesis of antibiotics. Our functional annotation of genes and their associated miRNAs might be helpful in understanding this targeted slicing on the pathogenesis of IgAN.

JUN and FOS are part of the transcription factor AP-1, which regulates the expression of genes involved in proliferation, cell death, differentiation, and inflammation (Durchdewald et al., 2009). During the last ten years, slew of studies has made it clear that IgAN occurs due to the overexpression of AGT in kidney (Takamatsu et al., 2008, Kobori et al., 2007, Nishiyama et al., 2011), but some recent studies in Japan and China have suggested that there has been no correlation among AGT and IgAN (Huang et al., 2010, Teranishi et al., 2015).

Fn1 is a non-collagenous glycoprotein and the principal component of the ECM. The previous study revealed that urinary FN excretion may be a sign of disease activity in IgAN (Roszkowska-Blaim et al., 2006). EGR1 is concerned with the proliferation and growth of cell (Hu et al., 2018). Early growth response factor 1 (Egr1) is a zinc-finger transcription factor expressed across different eukaryotic cells. Upregulation of Egr1 is associated with renal fibrosis and inflammation, especially in the development of diabetic nephropathy. Its role in the development of IgAN is not completely clear (Mohamad et al., 2018). has-miR-144-3p is involved in various biological function such as, it encourages the cell to prevent the apoptosis, PTEN inhibition, and maintain the activation of NF-κB, (Zhang et al., 2013). From recent studies, it have been investigated that the expression level of has-miR-144-3p is particularly high in IgAN patients and also considered as non-invasive biomarkers in IgAN (Duan et al., 2017).

Identification of aberrant pathways in IgAN patient might help to identify the molecular mechanism underlying and to uncover more enthralling and promising molecular candidates with effective diagnostic and prognostic value. These findings shed light light on the pathogenesis of IgAN and facilitate the development of personalized treatment. The disturbed pathways identified using integrated bioinformatics analysis may have important role to play in the pathogenesis of IgAN. Additional studied is required to investigate the molecular mechanisms of behind these aberrant pathways and IgAN development. Furthermore, due to a lack of experimental studies and verifications, we could not further explore how hub gene-miRNAs networks have effects on the diagnosis and therapy of IgAN in depth. Despite these limitations, this study may provide more accurate results based on integrated bioinformatic analysis compared to the single dataset studies.

## 5 Conclusion

In summary, a network among miRNA and hub genes were created which led to the identification of total 9 genes and 1 miRNA. Nine genes named FN1, EGR1, FOS, JUN, SERPINE1, MMP2, ATF3, MYC, and IL1B while hsa-miR-144-3p were considered as potential biomarker for IgAN. These hub genes and key miRNA might have a potential role in the origination and development of IgAN. Furthermore, a detailed study is needed for the understanding of complex molecular mechanisms of IgAN. In near future, further study and clinical trials are required for the identification of genes and key miRNAs having effective diagnostic and prognostic value, respectively. Our research will serve as a significant pioneer for the researchers who want to identify the associated pathways involved in the pathogenesis of IgAN. Based on the novel key miRNAs, experimental models may be designed in terms for the detection of pathogenesis, evaluation of risk, and in determining the targeted therapies of IgAN.

## CRediT authorship contribution statement

**Fatima Noor:** Formal analysis. **Muhammad Hamzah Saleem:** . **Muhammad Farhan Aslam:** Formal analysis. **Ajaz Ahmad:** . **Sidra Aslam:** Conceptualization, Methodology.

## Declaration of Competing Interest

The authors declare that they have no known competing financial interests or personal relationships that could have appeared to influence the work reported in this paper.

## References

[b0005] Barratt J., Feehally J. (2006). Treatment of IgA nephropathy. Kidnet Int..

[b0010] Barrett T., Wilhite S.E., Ledoux P., Evangelista C., Kim I.F., Tomashevsky M. (2012). NCBI GEO: archive for functional genomics data sets—update. Nucleic Acids Res..

[b0015] Bartels C.L., Tsongalis G.J. (2009). MicroRNAs: novel biomarkers for human cancer. Clin. Chem..

[b0020] Berger J. (1968). Intercapillary deposits of IgA-IgG. J. Urol. (Paris).

[b0025] Cline M.S., Smoot M., Cerami E., Kuchinsky A., Landys N., Workman C. (2007). Integration of biological networks and gene expression data using Cytoscape. Nat. Protoc..

[b0030] Coppo R., Robert T. (2020). IgA nephropathy in children and in adults: two separate entities or the same disease?. J. Nephrol..

[b0035] D'Amico G. (1988). Clinical features and natural history in adults with IgA nephropathy. Am J. Kidney Dis..

[b0040] Duan Z.-Y., Cai G-y, Li J.-J., Bu R., Chen X.-M. (2017). Urinary erythrocyte-derived miRNAs: emerging role in IgA nephropathy. Kidney Blood Press. Res..

[b0045] Durchdewald M., Angel P., Hess J. (2009). The transcription factor Fos, a Janus-type regulator in health and disease. Histol. Histopathol..

[b0055] Hennino M.-F., Buob D., Van der Hauwaert C., Gnemmi V., Jomaa Z., Pottier N. (2016). miR-21-5p renal expression is associated with fibrosis and renal survival in patients with IgA nephropathy. Sci. Rep..

[b0060] Hu F., Xue M., Li Y., Jia Y.-J., Zheng Z.-J., Yang Y.-L. (2018). Early growth response 1 (Egr1) is a transcriptional activator of NOX4 in oxidative stress of diabetic kidney disease. J. Diabetes Res..

[b0065] Hu S.-L., Wang D., Yuan F.-L., Lei Q.-F., Zhang Y., Cheng J.-Z. (2020). Identification of key genes and pathways in IgA nephropathy using bioinformatics analysis. Medicine (Baltimore).

[b0070] Huang H.-d., Lin F-j, Li X-j, Wang L-r, Jiang G-r (2010). Genetic polymorphisms of the renin-angiotensin-aldosterone system in Chinese patients with end-stage renal disease secondary to IgA nephropathy. Chin. Med. J. (Engl)..

[b0050] Jarrick S., Lundberg S., Welander A., Carrero J.J., Höijer J., Bottai M. (2017). Mortality in IgA nephropathy: a nationwide population-based cohort study. J. Am. Soc. Nephrol..

[b0075] Kobori H., Katsurada A., Ozawa Y., Satou R., Miyata K., Hase N. (2007). Enhanced intrarenal oxidative stress and angiotensinogen in IgA nephropathy patients. Biochem. Biophys. Res. Commun..

[b0080] Leung J.C., Chan L.Y., Saleem M., Mathieson P., Tang S.C., Lai K.N. (2015). Combined blockade of angiotensin II and prorenin receptors ameliorates podocytic apoptosis induced by IgA-activated mesangial cells. Apoptosis.

[b0085] Liang S., Jin J., Lin B., Gong J., Li Y., He Q. (2017). Rapamycin induces autophagy and reduces the apoptosis of podocytes under a stimulated condition of immunoglobulin a nephropathy. Kidney Blood Press. Res..

[b0090] Lim C., Kim Y., Chae D., Ahn C., Han J., Kim S. (2005). Association of C-509T and T869C polymorphisms of transforming growth factor-β 1 gene with susceptibility to and progression of IgA nephropathy. Clin. Nephrol..

[b0095] McGrogan A., Franssen C.F., de Vries C.S. (2011). The incidence of primary glomerulonephritis worldwide: a systematic review of the literature. Nephrol. Dial Transplant..

[b0100] Mering C.V., Huynen M., Jaeggi D., Schmidt S., Bork P., Snel B. (2003). STRING: a database of predicted functional associations between proteins. Nucleic Acids Res..

[b0105] Mohamad T., Kazim N., Adhikari A., Davie J.K. (2018). EGR1 interacts with TBX2 and functions as a tumor suppressor in rhabdomyosarcoma. Oncotarget.

[b0110] Nishiyama A., Konishi Y., Ohashi N., Morikawa T., Urushihara M., Maeda I. (2011). Urinary angiotensinogen reflects the activity of intrarenal renin–angiotensin system in patients with IgA nephropathy. Nephrol. DialTransplant..

[b0115] Qian W., Xiaoyi W., Zi Y. (2019). Screening and bioinformatics analysis of IgA nephropathy gene based on GEO databases. BioMed Res. Int..

[b0120] Rauen T., Floege J. (2017). Inflammation in IgA nephropathy. Pediatr. Nephrol..

[b0125] Ritchie, M.E., Phipson, B., Wu, D., Hu, Y., Law, C.W., Shi, W., et al., 2015. limma powers differential expression analyses for RNA-sequencing and microarray studies. Nucleic Acids Res. 43(7), e47. https://doi.org/10.1093/nar/gkv007. 10.1093/nar/gkv007 PMC4402510 25605792

[b0130] Rops A.L., Jansen E., van der Schaaf A., Pieterse E., Rother N., Hofstra J. (2018). Interleukin-6 is essential for glomerular immunoglobulin A deposition and the development of renal pathology in Cd37-deficient mice. Kidney Int..

[b0135] Rodrigues J.C., Haas M., Reich H.N. (2017). IgA nephropathy. Clin. J. Am. Soc. Nephrol..

[b0140] Roszkowska-Blaim M., Mizerska-Wasiak M., Bartłomiejczyk I. (2006). Urinary fibronectin excretion as a marker of disease activity in children with IgA nephropathy and Henoch-Schönlein nephropathy. Przegl Lek.

[b0145] Schena F.P. (1990). A retrospective analysis of the natural history of primary IgA nephropathy worldwide. Am. J. Med..

[b0150] Shannon P., Markiel A., Ozier O., Baliga N.S., Wang J.T., Ramage D. (2003). Cytoscape: a software environment for integrated models of biomolecular interaction networks. Genome Res..

[b0155] Takamatsu M., Urushihara M., Kondo S., Shimizu M., Morioka T., Oite T. (2008). Glomerular angiotensinogen protein is enhanced in pediatric IgA nephropathy. Pediatr. Nephrol..

[b0160] Tanaka K., Sugiyama H., Yamanari T., Mise K., Morinaga H., Kitagawa M. (2018). Renal expression of trefoil factor 3 mRNA in association with tubulointerstitial fibrosis in IgA nephropathy. Nephrology.

[b0165] Teranishi, J., Yamamoto, R., Nagasawa, Y., Shoji, T., Iwatani, H., Okada, N., et al., 2015. ACE insertion/deletion polymorphism (rs1799752) modifies the renoprotective effect of renin-angiotensin system blockade in patients with IgA nephropathy. J. Renin Angiotensin Aldosterone Syst. 16(3), 633–641. https://doi.org/10.1177%2F1470320313515036. 10.1177/1470320313515036 24452035

[b0170] Wyatt R.J., Julian B.A. (2013). IgA nephropathy. New Engl. J. Med..

[b0175] Xiao C., Rajewsky K.J.C. (2009). MicroRNA control in the immune system: basic principles. Cell.

[b0180] Yating, G., Meiling, S., Song, J., Xiong, Z., Hou, S., 2017. Meta-analysis of the relevance between Megsin rs1055901, rs1055902 and rs2689399 polymorphism and susceptibility of IgA nephrology in Asian population. Chongqing Med. 46(5), 648–650,653.

[b0185] Yu G.Z., Guo L., Dong J.F., Shi S.F., Liu L., Wang J.W. (2020). Persistent hematuria and kidney disease progression in IgA nephropathy: a cohort study. Am. J. Kidney Dis..

[b0190] Zhang B., Pan X., Wang Q., Cobb G.P., Anderson T.A. (2006). Computational identification of microRNAs and their targets. Comput. Biol. Chem..

[b0195] Zhang L.-Y., Ho-Fun Lee V., Wong A.M.G., Kwong D.L.-W., Zhu Y.-H., Dong S.-S. (2013). MicroRNA-144 promotes cell proliferation, migration and invasion in nasopharyngeal carcinoma through repression of PTEN. Carcinogenesis.

[b0200] Zhang Y., Yan X., Zhao T., Xu Q., Peng Q., Hu R. (2017). Targeting C3a/C5a receptors inhibits human mesangial cell proliferation and alleviates immunoglobulin A nephropathy in mice. Clin. Exp. Immunol..

